# Supplementary material for: Antidepressant Prescribing Trends for Adults With and Without Autism in the United Kingdom From 1997 to 2023: A Population‐Based Cohort Study Using Clinical Practice Research Datalink Aurum
Source: Autism Res. 2026 Aug 27;19(10):e70360. doi: 10.1002/aur.70360 (PMC13674362; doi:10.1002/aur.70360)
Supplement: Supplementary file 2 [file AUR-19-0-s001.zip › SadikSupCaptions.docx]

# Captions for supplementary tables and figures

Supplementary tables

ST1: Antidepressants included, plausible dose ranges in milligrams and class. SSRI = serotonin and noradrenaline reuptake inhibitor; TCA = tricyclic antidepressant; MAO-I = monamine-oxidase inhibitor.

ST2: Characteristics of patients with full-year follow-up in 1997, 2010 and 2023, including all adults under 65 years old, stratified by autism status. ADHD = attention deficit hyperactivity disorder; Psych contact = contact, referral or signposting to to a mental health service.

ST3: Characteristics of autistic groups and matched comparator groups. ADHD = attention deficit hyperactivity disorder; Psych contact = contact, referral or signposting to to a mental health service; IQR = interquartile range.

ST4: Characteristics of patients in autistic and matched groups with full-year follow-up in 1997, 2010 and 2023, including adults under 65 years old. ADHD = attention deficit hyperactivity disorder; Psych contact = contact, referral or signposting to to a mental health service.

ST5: Annual prescribing results

ST6: Lifetime prescribing results

ST7: New prescribing results

ST8: Annual class-level prescribing results

ST9: Annual substance-level prescribing results

ST10: Indication results

ST11: Mean dose results

ST12: Median dose counts used to calculate median dose results

ST13: Mean dose results, excluding new users

ST14: Counts used for median dose results, excluding new users

ST15: Duration results, survival method

ST16: Duration results, proportion of patients covered method.

Supplementary figures

Figure S1 : Participant flowchart

*Figure S1*

Figure S2 : Annual proportion of people prescribed each class of antidepressant by autism status. SSRI = serotonin-selective reuptake inhibitor; SSRI = serotonin and noradrenaline reuptake inhibitor; TCA = tricyclic antidepressant; MAO-I = monoamine-oxidase inhibitor. Shaded areas represent 95% confidence intervals.

*Figure S2*

Figure S3 : Proportion of patients taking antidepressants at 1 year, 2 years and 3 years after starting an antidepressant, by calendar year and autism status. Shaded areas represent 95% confidence intervals.

*Figure S3*

Figure S4 : Mean doses of citalopram, fluoxetine and sertraline prescribed in each calendar year, by autism status, after excluding patient-years in which antidepressants were being initiated. Shaded areas represent 95% confidence intervals.

*Figure S4*

Figure S5 : Median doses of citalopram, fluoxetine and sertraline prescribed in each calendar year, by autism status. Shaded areas represent interquartile range.

*Figure S5*

Figure S6 : Median doses of citalopram, fluoxetine and sertraline prescribed in each calendar year, by autism status, after excluding patient-years in which antidepressants were being initiated. Shaded areas represent interquartile range.

*Figure S6*

Figure S7: Antidepressant prescribing patterns for autistic adults with intellectual disability (ID), autistic adults without ID and unmatched non-autistic adults, by sex. Top row: proportion of adults prescribed antidepressants in each calendar year by autism status - any prescription in the current calendar year. Second row: Top row: proportion of adults prescribed antidepressants in each calendar year by autism status - new prescriptions among those without prescriptions in prior calendar year. Third & forth rows: proportion of adults starting an SSRI who have anxiety or depression recorded in the two months either side, by calendar year and autism status. Fifth row: Mean dose of sertraline prescribed in each calendar year, by autism status. Bottom row: Proportion of antidepressant courses lasting at least 2 years, by year of course initation and autism status. Shaded areas represent 95% confidence intervals.

*Figure S7*

Figure S8: Antidepressant prescribing patterns for autistic adults with intellectual disability (ID), autistic adults without ID and unmatched non-autistic adults, by age-group. Top row: proportion of adults prescribed antidepressants in each calendar year by autism status - any prescription in the current calendar year. Second row: Top row: proportion of adults prescribed antidepressants in each calendar year by autism status - new prescriptions among those without prescriptions in prior calendar year. Third & forth rows: proportion of adults starting an SSRI who have anxiety or depression recorded in the two months either side, by calendar year and autism status. Fifth row: Mean dose of sertraline prescribed in each calendar year, by autism status. Bottom row: Proportion of antidepressant courses lasting at least 2 years, by year of course initation and autism status. Shaded areas represent 95% confidence intervals.

*Figure S8*
